# Shaping transcriptional responses to a phytohormone

**DOI:** 10.1038/s42003-023-04440-x

**Published:** 2023-01-13

**Authors:** David S. Favero

**Affiliations:** Communications Biology, https://www.nature.com/commsbio/

## Abstract

The BIL1/BZR1 transcription factor is known to regulate transcriptional responses to the brassinosteroid class of phytohormones by directly recognizing short cis regulatory elements in promoters. A new study by Shohei Nosaki, Nobutaka Mitsuda, and colleagues published in *Nature Plants* indicates that binding of this transcription factor is additionally affected by nucleobases that influence DNA shape but are not directly contacted by BIL1/BZR1.

Brassinosteroids (BR) play key roles promoting growth in plants and are also involved in the regulation of other developmental processes as well as various stress responses. Two transcription factors (TF), BIL1/BZR1 and BES1, are predominantly responsible for altering gene transcription in response to BR. It is known that tight binding of BIL1/BZR1 to DNA is dependent on direct recognition of the nucleobases contained in a short cis regulatory element, 5′-CGTG-3, by residues in the TF’s DNA-binding domain^1,2^. Beyond this, however, the promoter features that facilitate binding of BIL1/BZR1 to BR-responsive genes are not well understood.

A recent study by Nosaki et al^3^. provides new insight into the DNA features that enable tight binding of BIL1/BZR1 to promoters in *Arabidopsis*. Through combined analysis of microarray and DAP-seq data, the authors identified the dinucleotides immediately upstream or downstream of the core 5′-NNCGTG-3′ nucleobases that are directly contacted by BIL1/BZR1 as likely to impact binding of the TF. An effect of these dinucleotides on promoter binding and consequently transcriptional repression by BIL1/BZR1 was subsequently confirmed using a combination of approaches, including transient effector-reporter experiments. Specifically, it was shown that the presence of a pyrimidine followed by a purine on either side of the core 5′-NNCGTG-3′ sequence generally favors binding of BIL1/BZR1. Additional analyses of BIL1/BZR1-DNA complexes suggested that although the TF does not directly contact the nucleobases of the dinucleotides found on either side of the core 5′-NNCGTG-3′ sequence, these nucleotides impact binding by affecting flexibility of the DNA. DNA with greater flexibility can form a more stable bond with BIL1/BZR1 because binding of the TF induces substantial distortion of the DNA backbone.

Nosaki and colleagues propose that an absence of base-stacking overlap between a pyrimidine followed by a purine promotes DNA flexibility. Consequently, this dinucleotide combination promotes stable binding of BIL1/BZR1 when it is located either directly before or after the core 5′-NNCGTG-3′ sequence.

In addition to providing new information about the molecular mechanisms that regulate transcriptional responses to BR in plants, these results provide novel insight into how nucleotide sequence can affect TF binding. Specifically, that nucleotides can demonstrably impact TF binding in the absence of direct contact between their nucleobases and residues in the DNA-binding domain of the TF, through an effect on DNA shape.
